# Recommendations for the use of Serious Games in people with Alzheimer's Disease, related disorders and frailty

**DOI:** 10.3389/fnagi.2014.00054

**Published:** 2014-03-24

**Authors:** Philippe H. Robert, Alexandra König, Hélene Amieva, Sandrine Andrieu, François Bremond, Roger Bullock, Mathieu Ceccaldi, Bruno Dubois, Serge Gauthier, Paul-Ariel Kenigsberg, Stéphane Nave, Jean M. Orgogozo, Julie Piano, Michel Benoit, Jacques Touchon, Bruno Vellas, Jerome Yesavage, Valeria Manera

**Affiliations:** ^1^EA CoBTeK/IA, University of Nice Sophia AntipolisNice, France; ^2^Centre Mémoire de Ressources et de Recherche, CHU de NiceNice, France; ^3^Alzheimer Centrum Limburg, School of Mental health and Neurosciences, Maastricht UniversityMaastricht, Netherlands; ^4^Centre INSERM U897-Epidemiology-Biostatistics, University of Bordeaux, ISPEDBordeaux, France; ^5^Inserm, UMR1027Toulouse, France; ^6^Université de Toulouse III, UMR1027Toulouse, France; ^7^CHU de Toulouse, Service d'épidémiologie et Santé PubliqueToulouse, France; ^8^INRIA - STARS - Sophia AntipolisFrance; ^9^Kingshill Research CentreSwindon, UK; ^10^Centre Mémoire de Ressources et de RechercheCHU de Marseille, France; ^11^CMRR CHU de Paris, IM2A, INSERM, UMR-S 975 (ICM)Paris, France; ^12^Hôpital La Salpêtrière, Université Pierre et Marie Curie-Paris 6Paris, France; ^13^McGill Centre for Studies in AgingVerdun, QC, Canada; ^14^Fondation Médéric AlzheimerParis, France; ^15^pRED, Neuroscience, Roche, Centre MémoireBasel, Switzerland; ^16^Centre Mémoire de Ressources et de Recherche, CHU de BordeauxBordeaux, France; ^17^Centre Mémoire de Ressources et de Recherche, CHU de MontpellierMontpellier, France; ^18^INSERM UMR 1027, Gerontopole, CHU ToulouseToulouse, France; ^19^INSERM UMR1027, Université de Toulouse III Paul SabatierToulouse, France; ^20^Palo Alto Veterans Affairs Health Care SystemPalo Alto, CA, USA; ^21^Department of Psychiatry and Behavioral Sciences, Stanford University School of Medicine, Stanford UniversityStanford, CA, USA

**Keywords:** Alzheimer's disease, mild cognitive impairment, frailty, serious games, recommendations, rehabilitation, SWOT analysis, non pharmacological treatment

## Abstract

Alzheimer's disease and other related disorders (ADRD) represent a major challenge for health care systems within the aging population. It is therefore important to develop better instruments to assess the disease severity and progression, as well as to improve its treatment, stimulation, and rehabilitation. This is the underlying idea for the development of Serious Games (SG). These are digital applications specially adapted for purposes other than entertaining; such as rehabilitation, training and education. Recently, there has been an increase of interest in the use of SG targeting patients with ADRD. However, this field is completely uncharted, and the clinical, ethical, economic and research impact of the employment of SG in these target populations has never been systematically addressed. The aim of this paper is to systematically analyze the Strengths, Weaknesses, Opportunities, and Threats (SWOT) of employing SG with patients with ADRD in order to provide practical recommendations for the development and use of SG in these populations. These analyses and recommendations were gathered, commented on and validated during a 2-round workshop in the context of the 2013 Clinical Trial of Alzheimer's Disease (CTAD) conference, and endorsed by stakeholders in the field. The results revealed that SG may offer very useful tools for professionals involved in the care of patients suffering from ADRD. However, more interdisciplinary work should be done in order to create SG specifically targeting these populations. Furthermore, in order to acquire more academic and professional credibility and acceptance, it will be necessary to invest more in research targeting efficacy and feasibility. Finally, the emerging ethical challenges should be considered a priority.

## Introduction

Due to the increasing average lifespan, the occurrence of neurodegenerative disorders such as dementia has risen by unprecedented levels, thus engendering high socio-economic costs. Alzheimer's disease (AD) is the most common type of dementia, and affects one in eight aged 65 and older (Alzheimer's Disease International, 2011). Prevalence studies estimate that the number of people affected will reach 81.1 million worldwide by 2040 (Ferri et al., 2005). As a consequence, the early detection and the treatment of AD and related disorders (ADRD) are considered as research priorities (Ballard et al., 2011).

In the last decades there has been a growing interest in employing Information and Communication Technologies (ICT) to help assess and evaluate patients' functional impairments, as well as to help and support patients in everyday activities (Wichers et al., 2011). Concerning clinical assessment, ICT play an important role allowing the development of new methods to evaluate more objectively behavioral and functional deficits (König et al., 2014). This is important for clinical activity, as well as for research purposes (Robert et al., 2013). But beyond being important for assessment, ICT can also play a key role in the patients' treatment, stimulation, and rehabilitation. This is the underlying idea for the development of Serious Games (SG), which are digital applications specialized for purposes other than entertaining, such as training and educating, informing, communicating, marketing, leading societal/ideological impact on specific subjects, or enhancing user's aptitudes or cognitive/physical functions.

The elderly population (above 50 years) represents now a considerable portion of digital gamers (e.g., 14% in Germany BIU, 2011 and 29% in USA ESA, 2011), which is predicted to increase. For this reason, SG may represent a low-barrier, motivating, sustainable and relatively cheap method to improve, or at least delay the onset of impairments in selected social, sensory-motor, and emotional functions (McCallum, 2012). Recently, some studies have started to investigate the effectiveness of SG in people with ADRD (McCallum and Boletsis, 2013), but the field is still completely uncharted, and the clinical, ethical, economic, and research impact of the employment of SG in these target populations has never been rigorously addressed and discussed.

The purpose of the present methods paper is to put together recommendations for the development and use of SG in patients with ADRD and frailty gathered from stakeholders in the field, and to analyze systematically the Strengths, Weaknesses, Opportunities and Threats (SWOT) of employing SG with these patients. We will now briefly describe the target population and review the results of the few studies that addressed the employment of SG in that area, before describing in more detail the aims of the present work.

### ADRD and frailty

AD is a neuro-degenerative disorder where memory functions are primarily affected at the early stages of the disease (Agüero-Torres et al., 1998). Persons with AD generally deteriorate progressively through multiple stages over several years. Despite some discrepancies, there is a growing consensus in subdividing the course of AD into three stages: (1) a preclinical/asymptomatic stage, only revealed by biomarker evidence; (2) a predementia phase, characterized by the impairment in memory or other cognitive domains not negatively affecting social and/or occupational functioning (also known as Mild Cognitive Impairment, MCI) (3) a dementia phase, in which cognitive disturbances significantly interfere with the capacity of independent living (Dubois et al., 2010). At this stage, cognitive symptoms are often associated to behavioral and psychological symptoms such as apathy or agitation (Aalten et al., 2003).

MCI is defined as a cognitive decline greater than expected for an individual's age, but which does not interfere notably with activities of daily life (Petersen et al., 1999; Gauthier et al., 2006). Some people with MCI remain stable or return to a normal state over time, but more than half progress to dementia within 5 years. Therefore, MCI can be regarded as a risk state for dementia and its early identification could offer opportunities for preventative interventions (Albert et al., 2011).

Another concept that has recently attracted the attention of researchers and clinicians is that of frailty, defined as a multidimensional geriatric syndrome characterized by increased vulnerability to stressors as a result of reduced capacity of different physiological systems (Kelaiditi et al., 2013). Traditionally, the concept of frailty has focused principally on the physical domain (Fried et al., 2001). Recent work has started to study more deeply the cognitive impairment due to physical frailty, and lead to the definition of cognitive frailty, defined by the simultaneous presence of both physical frailty and cognitive impairment without the presence of a concomitant neurological disease (see Kelaiditi et al., 2013 for a review). Cognitive frailty is viewed as a potential precursor of neurodegenerative processes with good potential for reversibility, and thus is the ideal target of early interventions.

### State of the art: the use of serious games with people with ADRD

There is evidence that SG can successfully be employed to train physical and cognitive abilities in elderly people (e.g., Anguera et al., 2013; see Wiemeyer and Kliem, 2012 for a review of the studies employing SG in prevention and rehabilitation of elderly people). Recently, some studies have started to investigate the effectiveness of SG in people with AD, MCI, and related disorders. McCallum and Boletsis (2013) performed a literature review of the experimental studies conducted to date on the use of SG in neurodegenerative disorders. In summary, the results of the 15 reported studies suggest that: (1) physical games (or exergames, i.e., games that promote physical fitness) can positively affect several health areas of the players with mild AD and MCI, such as balance and gait (Padala et al., 2012), and voluntary motor control (Legouverneur et al., 2011); (2) cognitive games (i.e., games which target cognitive improvement) can improve a number of cognitive functions, such as attention and memory (Stavros et al., 2010; Weybright et al., 2010; Rosen et al., 2011) and visuo-spatial abilities (Yamaguchi et al., 2011); (3) both physical and cognitive games can have a positive impact on social and emotional functions, for instance they can improve the mood and increase positive affect and sociability (Weybright et al., 2010; Boulay et al., 2011; Yamaguchi et al., 2011) and reduce depression (Férnandez-Calvo et al., 2011). Very few studies investigated the effects of the use of games for social/emotional health (which encourage the players to link with their friends and/or improve their social and emotional life) in dementia, but the results are encouraging (Boulay et al., 2011).

Despite these promising results, a number of studies showed that elderly people and people with ADRD have problems in using many of the SG currently available on the market. Their difficulties include problems in getting familiar with the game technology and embarrassment about using the tools designed for the game (e.g., Wollersheim et al., 2010; Legouverneur et al., 2011). Furthermore, certain games were considered too demanding or even risky for elderly people (e.g., Sohnsmeyer et al., 2010). These difficulties derive from the fact that most of the SG currently employed have been developed for entertainment purposes (e.g., the Nintendo Wii Fit, Wii Sports, and Big Brain Academy) and with a “typical healthy user” in mind.

To overcome this problem, SG targeting specifically ADRD are starting to emerge (e.g., Benveniste et al., 2010; Nor Wan Shamsuddin et al., 2011; Tarnanas et al., 2013), along with guidelines ensuring their usability among the targeted populations (e.g., Bouchard et al., 2012; Fua et al., 2013). However, these recommendations are still very sparse.

### Aims of the present work

As outlined in the previous paragraphs, there is an increasing interest in employing SG with patients with frailty and ADRD, and a general feeling that these cutting-edge applications may open new avenues for clinical treatment and experimental investigation of important issues. However, the field is completely uncharted. Strong evidence concerning the effectiveness of SG as clinical tools is still missing, together with a consensus on how, when and for what purposes SG should be employed. Furthermore, the ethical, social, and economic consequences of the employment of SG in these clinical populations have never been systematically investigated. As the topic is very new and relatively few scientific papers have been published so far, we believe that it would benefit from a structured dialog and discussion between different stakeholders: including people working in the health domain (e.g., clinicians, neuropsychologists, geriatricians, etc.), people working on ICT (e.g., engineers and SG designers), people working in the business domain (e.g., product marketing, business development), as well as patients and their caregivers (e.g., people working on nursing homes, family caregivers, etc.).

Starting from these considerations, we organized a two-round workshop (IA workshop) with stakeholders in the field with the aims of (a) analyzing systematically the employment of SG in frailty and ADRD (SWOT analysis), and (b) gathering recommendations for the development and use of SG targeting these populations.

## Methods

### Workshop strategy

The analyses and recommendations reported in the present paper were collected and discussed during the workshop “Innovation Alzheimer 2013” (IA workshop 2013), organized by the CoBTek (Cognition—Behavior—Technology) Research Unit of the University of Nice—Sophia Antipolis (UNS) in Nice, France. CoBTek's main mission is to use ICT, particularly imaging and video analytic techniques to: (1) Improve diagnostics and treatment of behavioral and cognitive symptoms in ADRD (2) Develop new strategies in order to prevent, help and assist elderly people (3) Improve autonomy in the elderly.

The IA workshop 2013 had a two-step design (two rounds plus a web survey).

### First round

The first round took place in Nice on November 7th, 2013, and involved 50 participants including health care professionals and family association representatives (*n* = 25), ICT engineers (*n* = 10), representatives of companies involved in ICT, and economical experts (*n* = 15). It started with a 3-h plenary session, where recent works employing SG in elderly people and people with ADRD were presented. The aim was to ensure that participants coming from different backgrounds were familiar with terms such ADRD, frailty, and SG. After the plenary session, participants were divided in three 2-h parallel sessions:

#### SWOT analysis session

Participants (2 groups of 4 participants) were presented with items concerning SWOT of SG in ADRD deriving from a former literature review, and asked to prioritize them, as well as to generate new ideas. The list of items proposed to the participants, as well as the new items that were proposed during the session, are reported in the Results section.

#### Serious game design session

Two groups of 4 participants worked separately to design a SG targeting patients suffering from ADRD.

#### Recommendation session

One group of 34 participants worked to generate practical recommendations for the development and use of SG in ADRD. Why, Where, When, What, and How questions were used as a guideline to structure the session. The list of the 6 questions asked to the participants is reported in Table 1, and included two general questions and four questions focused on the use of SG in patients with ADRD. First, participants were presented with the questions, and asked to respond to them through a brainstorming carried out in small groups. After the brainstorming, participants were presented with a list of responses to each question, and were asked to rate the importance of each item on a 0–3 scale (0 = not important at all/not adapted at all; 1 = not very important/not very adapted; 2 = important/adapted; 3 = very important/very adapted).

**Table 1:** **Questions for recommendation session**.

**General questions**
SG for *whom*? i.e., what should be the target population for SG?
SG for *what*? i.e., *Wh*y is it interesting to use the SG?
**Questions focused on dementia-related disorders**
*Why* should SG be employed with patients with ADRD? *What* is the clinical target?
*When* (how frequently) should SG be used in patients with ADRD?
*Where* should SG be used for patients with ADRD
*Whom* should patients with ADRD play SG *with*?

After voting, participants were presented with a list of practical recommendations for the development of SG adapted to people with ADRD that emerged from a previous literature review, and were asked to comment them and to generate new ideas. The complete list of items proposed to the participants is reported in the Results section.

At the end of the three parallel sessions, all participants were involved in a 1-h plenary discussion, where all the groups' works were presented and commented.

### Web survey

Based on the results collected from the recommendation session during the first round, a web survey was proposed to all CNR members, representatives of the French Memory centers, and members of the European FP7 project VERVE. 30 experts completed the survey, including health care professionals (*N* = 17), representatives of companies involved in ICT and economical experts (*n* = 6), ICT engineers (*n* = 1), and researchers (*N* = 6). Participants were presented with the same list of questions and items used during the first round, and asked to rate the importance of each item on a 0–3 scale (0 = not important at all/not adapted at all; 1 = not very important/not very adapted; 2 = important/adapted; 3 = very important/very adapted).

### Second round

The second round took place on November 15th during the 6th edition of Clinical Trials on Alzheimer's Disease (CTAD) conference held in San Diego (November 14–16th, 2013). The Consensus Group included 10 clinicians (geriatricians, epidemiologists, neurologists, psychiatrists, psychologists), 1 ICT engineer, and 1 representative from the pharmacological industry.

The week of the second round participants received a first draft of the recommendations. During the second round, the CoBTeK team presented the preliminary results collected during the first round and the web survey, and asked for comment on these results within a group discussion, which was audio recorded. The objective was to validate the manuscript draft, and to collect new and/or different ideas coming from international experts.

## Results

### SWOT analysis

Participants of the SWOT analysis session (round 1) were presented with items concerning SWOT of the use of SG in people with ADRD based a literature review (e.g., Rizzo and Kim, 2005), and asked to comment and prioritize the items, as well as to generate new ideas. The reported results (summarized in Table 2) include the findings that emerged from the SWOT analysis session of round 1, commented on and integrated by all the participants of the first round during the general discussion, and by the participants of the consensus group during the second round.

**Table 2:** **Summary of a SWOT analysis of the use of SG in ADRD**.

**Strengths**	**Weaknesses**
Interface adapted to the user	Interface challenges
Gaming factors to enhance motivation, positive mood and improve assessment	Non-naturalistic interactions
	Wires and displays
Independent practice and self-assessment	Immature engineering process
Safe testing and training environment	Expensive equipment
Promoting social bonding	Poor platform compatibility
Enhanced ecological validity	Software difficult to use
Control of stimulus delivery	Lack of generalization
Cuing stimuli for error-free learning	Addiction
Performance analysis in real time	Side effects
Real-time feedback delivery	
Promoting learning processes	
Low-cost, duplicable environments	
**Opportunities**	**Threats**
Emerging advances in technology	Ethical challenges
Real time data analysis	Poor integration with the clinical practice
Gaming industry drivers	Lack of assessment methodology
Intuitive appeal to the public	Lack of feasibility and efficacy studies
New professions	Lack of regulation
Closeness between scientific, technical, and clinical communities	Lack of business model
	Too few cost/benefit proofs
SG as research instruments	Technological vs. clinical tool
Telerehabilitation	Aftereffects
Big market	The perception that the technological tools will eliminate the need for the clinician
	Unrealistic expectations
	Academic and professional acceptance
	Technophobia

#### Strengths

***Interface adapted to the user***. In the context of SG it is possible to create game interfaces adapted to the users' capacities and interests. Motivation is a crucial aspect for the success of rehabilitation programs, but differs from one person to another (Leone et al., 2012). One of the advantages of SG is that it is possible to generate games with different story-plots (e.g., cooking, travelling), but targeting the same ability (e.g., physical activity and executive functions). Furthermore, each SG can be potentially individualized. For example users may be able to generate personalized avatars, and the game can incorporate pictures or videos of familiar objects and environments. Adaptation is important also when it comes to the users' impairments. For instance, people with visual problems may receive auditory cues instead of visual cues, and people with memory problems may be prompted with repeated instructional cues. Taking into account the users' impairments is particularly important when targeting people with dementia-related disorders.

***Gaming factors to enhance motivation, positive mood and improve assessment***. SG have a playful character, which is supposed to enhance motivation and to improve the users' mood; an aspect which is particularly important when targeting clinical populations. Gaming factors can also improve assessment abilities. Plato said, “You can discover more about a person in an hour of play than in a year of conversation.” Observing and quantifying a person's behavior when participating in a SG may provide information even more reliable than those acquired with traditional performance assessments, because the person engaged in a gaming task is less focused on the fact of being “tested,” which is usually reported as very stressful (Cassady and Johnson, 2002).

***Independent practice and self-assessment***. SG, due to their “light” interfaces, their playful aspect and interactivity, and their high level of immersion (for the VR-based SG) can be employed for self-assessment and home-based skill practice. These are common components of most rehabilitation programs, considered crucial to promote activity automation, and generalization of the learned skills to every-day behavior. The possibility to carry out an autonomous activity can also lead to mood improvements and self-esteem enhancement. Furthermore, the home-based and independent practice would contribute to reduce the costs sustained by insurances and by the public healthcare system.

***Safe testing and training environment***. When developing ecological assessment and rehabilitation instruments, it is necessary to consider safety risks (for instance, the risks due to driving errors, but also the risks of burning oneself with a hot pan). SG represent an optimal solution to this problem, minimizing these kinds of risks.

***Promoting social bonding***. Social isolation and lack of social interactions are often reported as crucial problems by elderly people and people with dementia-related disorders (Cattan et al., 2005). SG may play a role in promoting social interactions. For instance, there are SG that can be played by multiple players physically co-present, or in groups. Some multi-player SG can be played online by people connected from remote locations. In this case the social interaction is more limited; even if some preliminary studies suggest that participants (undergraduate students) perceive the interactions developed during online SG as good quality social interactions (Mansour and El-Said, 2008), the prevalence and extent of social activities in online group games are intrinsically different from real world social interactions (e.g., Ducheneaut et al., 2006).

***Enhanced ecological validity***. Traditional clinical (non-pharmacological) rehabilitation methods have been criticized for their limited ecological validity (Chaytor and Schmitter-Edgecombe, 2003). The same concern applies to diagnostic laboratory testing. An important strength of SG, especially of those based on VR, is that they can offer rehabilitation environments which simulate real life environments and activities.

***Control of stimulus delivery***. Another strength of SG (and of all the tests and rehabilitation programs involving ICTs) compared to traditional rehabilitation methods is the possibility to systematically control and deliver stimuli, which represents a basic foundation of all human research.

***Cuing stimuli for error-free learning***. This dynamic stimulus delivery and control also allows the presentation of cuing stimuli that could be used for “error-free” learning and similar approaches.

***Performance analysis in real time***. The evaluation of patient performance typically involves a *post-hoc* examination of numeric data and subsequent translation of that information into graphic representations. Videotapes of the activities have been used to provide a more naturalistic performance evaluation, but it is often difficult to correlate these observations to the other numerical data. SG offer the therapists (and caregivers) the possibility to record and visualize the activity immediately after it has been recorded, and to measure the patient performance in real-time.

***Real-time feedback delivery***. In the context of SG, it is possible to provide feedback on performance and on accomplished activities, which is considered a key element to improving learning and rehabilitation of functional activities.

***Promoting learning processes***. When thinking about elderly people and people with dementia, there is a tendency to focus on rehabilitation and recovery of lost functions. However, neuroplasticity can also be improved by learning new things and activities, and by promoting positive affect and stress reduction (Davidson and McEwen, 2012). SG can be easily employed for this purpose. The employment of ICT when playing a SG represents a learning opportunity and challenge for elderly people and people with dementia-related disorders. Furthermore, SG has been shown to be able to improve mood and to decrease stress (Russoniello et al., 2009).

***Low-cost duplicable environments***. Contrary to traditional rehabilitation methods that usually rely on costly physical mock-ups owned by specialized centers, SG offer the capacity to produce and distribute cheap and identical “standardized” environments. Within such digital rehabilitation scenarios, normative data can be accumulated for performance comparisons needed for diagnostics, research and for training purposes.

#### Weaknesses

***Interface challenge I: non-naturalistic interactions***. Current SG platforms are still limited in the degree to which they allow users to naturalistically interact with them. Progress has been made, but it is necessary to invest more in usability testing, especially when designing SG for elderly and fragile people. Many elderly people are not used to interacting with high-tech interfaces, and the initial approach with SG may cause them a high cognitive and affective load, which may significantly slow down the learning curve. It is necessary to avoid any extra, non-automatic cognitive effort that could represent a distraction for the patient, and thus limit the assessment and rehabilitation processes.

***The interface challenge 2: wires and displays***. In order to use SG on ordinary PC/laptops or televisions, it is often necessary for the user to connect a number of cables and modify the setups. This may be too difficult for non-expert users, especially for elderly, and fragile people.

***Immature engineering process***. Building, testing, and maintaining a rehabilitation system based on SG is a very complex process, and there is a lack of a standard methodology. Developers must integrate disparate knowledge in both engineering and rehabilitation that include sub-areas such as tracking, displays, interaction, computer graphics, simulation, human factors, biokinesiology, cognitive psychology, and so forth.

***Expensive equipments***. To be effective, some VR-based SG need fully immersive projection displays (i.e., CAVE, Powerwalls, Immersadesks), which are very expensive, and thus not easy to install where the assessment/rehabilitation programs usually take place (hospitals, day clinics, etc.).

***Poor platform compatibility***. Most of SG are not inter-operable, and the applications are not written in a simple and reconfigurable manner. This represents an important problem, especially because the target places where the SG should be used (e.g., hospitals, nursing homes) are equipped with disparate (often not regularly updated) systems. Furthermore, many SG applications require a fast and reliable internet connection, which is sometimes not available, or not optimized.

***Software difficult to use***. Healthcare professionals are not programmers. Consequently, in order to maximize the usability and usefulness of rehabilitation program based on SG, great care needs to be placed on building an intuitive front-end interface. Similarly, the applications used to visualize performance results should be intuitive and easy to use. These remarks also apply to home-based SG managed directly by the patients and their caregivers.

***Lack of generalization***. Although SG try to promote ecological interactions and to create naturalistic environments, it is difficult to generalize the skills learned during SG to the real life context. For instance, learning to recognize other peoples' emotions from facial expressions in a SG does not guarantee that the user will actually demonstrate an improved ability to recognize emotions via facial expressions in a real social interaction context.

***Addiction***. Like many digital games, SG could cause addiction (Griffiths and Davies, 2002). Even if SG have mainly training purposes (e.g., enhancing memory and attention), people may spend too much time playing, and reduce the amount of time they dedicate to other activities (e.g., physical activity, hygiene, etc.).

***Side effects***. Side effects can be observed especially in the VR-based SG (Cobb et al., 1999). Cybersickness is a form of motion sickness with symptoms reported to include nausea, vomiting, eyestrain, disorientation, ataxia, and vertigo. After effects may also include symptoms such as disturbed locomotion, changes in postural control, perceptual-motor disturbances, flashbacks, drowsiness, fatigue, and generally lowered arousal. When using simpler SG (not VR-based), after effects may include headaches due to prolonged fixation of the display. In order to obtain usable and safe SG for fragile people, it is necessary to adapt them to the specific patients' deficits and problems.

#### Opportunities

***Emerging advances in technology***. Progress in the Graphics/Video Integration brings more visual realism to SG, representing an important component for developing effective rehabilitation programs. Another positive development is the trend toward wireless technology, which reduces the need for complex wire configurations. Similarly, due to the increase of demands in the consumer market, display devices are becoming cheaper. For VR-based SG, autostereoscopy makes stereo viewing possible without the need to wear any special equipment (i.e., polarized or active shutter-type glasses), a feature that can enhance the overall usability of VR systems.

***Real-time data analysis***. One of the strengths of rehabilitation programs based on SG is the possibility to collect and analyze performance data in real-time, and to provide the user with rapid performance feedback. This is possible in the context of cognitive tasks (e.g., providing results of memory or attention based games), but as well in the domain of motor tasks, thanks to the emergence of real-time motion analysis coupled with direct acquisition of motion data.

***Gaming industry drivers*** The recent growth in the interactive gaming-industry area, and its interest in the domain of health and ageing, will continue to drive the development of products targeting specific populations, such as people with dementia-related disorders.

***Intuitive appeal to the public***. The general public is very attracted to the idea of using SG with therapeutic or prevention targets. This interest is increased by the attention devoted to this domain by the media.

***New professions***. In order to create, develop, test and sell SG targeting specific populations, such as people with dementia-related disorders, it is necessary to rely on multi-disciplinary teams including people with different backgrounds and abilities, such as engineers, healthcare professionals, and businessmen. This process will lead to the creation of new professions (e.g., engineers with a deeper knowledge of specific health-related issues), and will represent an economical opportunity to create new job positions.

***Closeness between scientific, technical, and clinical communities***. The interdisciplinary work between the communities of researchers, engineers and clinicians that focus on SG for rehabilitation purposes are becoming more frequent.

***SG as research instruments***. SG can improve our knowledge of brain functioning. For instance, they can be easily used in association with brain imaging techniques, as they allow to interact in complex environments almost without moving (e.g., using a joystick). This feature of SG explains the increase of funding dedicated to projects that try to integrate the use of SG in domains such as neuropsychology and behavioral neuroscience. SG may also be employed as motivating factors to facilitate participants' recruitment, and to reduce the incidence of drop-outs.

***Telerehabilitation***. Due to the flexibility of the Internet, the idea of delivering rehabilitation and therapy to patients in remote locations has been a popular topic. The application of SG within a tele-rehabilitation format is the next logical step, allowing a therapist to check the utilization and progress of patients, or to manually modify the game parameters. Similarly, this would allow technicians to perform maintenance operations without the need to go to the users' home.

***Big market***. The number of people with dementia-related disorders is very high and destined to increase, and thus the tools developed to assess and rehabilitate dementia-related impairments are potentially addressing a large number of users. This makes the market interesting for high-tech companies, as well as for insurance companies.

#### Threats

***Ethical challenges***. Professionals must carefully consider and address incumbent ethical threats concerning the use of SG with fragile people, and the fact that the immersion in an environment too realistic may sometimes cause more problems than benefits. Also, questions concerning the disclosure of private personal information in the domain of internet-based SG should be addressed carefully.

***Poor integration with the clinical practice***. Although SG can be employed as clinical tools, they are poorly integrated in standard clinical and neuropsychological practice. In order to be effective, SG should be used only after a standard clinical and neuropsychological assessment of patient's deficits and abilities has been performed. This standardized evaluation (independent of the evaluation provided by SG) should be used to decide which SG should be employed with a specific patient, and with which level of difficulty.

***Lack of assessment methodology***. The field lacks standard methodologies to verify the effectiveness and efficacy of the use of SG. In order to understand if SG achieve the participants objectives, it is necessary to establish rigorous methods to assess the users' performance improvements, based on the methods used for clinical assessment. Clinicians and therapists should be involved directly in the assessment stage, especially during feasibility studies.

***Lack of feasibility and efficacy studies***. The interest in employing SG with patients with ADRD emerged only recently (see McCallum and Boletsis, 2013), and as a consequence the field is completely uncharted. Rigorous feasibility and efficacy studies will be necessary to develop a better understanding of the physical, psychological, and social effects and consequences of employing SG in these populations, and to predict and anticipate possible adverse consequences.

***Lack of regulation***. The field lacks regulations concerning the development, marketing and use of SG with sensitive populations, such as people with dementia-related disorders. It is extremely important to create standard rules for companies and institutions that create, test, sell and use SG, in order to guarantee that SG are safe, useful, and respect ethical principles. This lack of regulations is responsible for some false promises made in the past through media advertisement.

***Lack of business model***. Most SG are designed with the aim to create a prototype, and not a product for the market. As a consequence, the business model behind SG is not clear (e.g., should SG be free for the final user? who should pay for them, and for their cost of development? how should copyright issues be managed?). This lack can delay investments that game industries and companies working on ICT would devote to SG applications.

***Too few cost/benefit proofs***. The field lacks definitive cost/benefit analyses. Such analyses must spell out both the clinical and economic benefits of SG, weighed against the costs for using them over already-existing traditional methods.

***Technological vs. clinical tools***. SG are often considered by the general public as interesting and engaging technological tools. However, SG targeting assessment and rehabilitation of dementia-related disorders should be considered primarily as clinical tools, and thus employed with the same care and cautions that apply to all clinical instruments.

***Side effects***. As discussed in the “Weaknesses” section, side effects (such as cybersickness, perceptual-motor disturbances, drowsiness, fatigue, and headaches) may occur in users after using SG applications. If these aftereffects are not understood and managed properly, there is the possibility that a developer, clinician, researcher, or supporting institution could be held responsible for them.

***The perception that the technological tools will eliminate the need for the clinician***. This issue belongs to the more general problem of using technology-based instruments in the health sector. Although supporters of new technologies point out that SG are simply tools that extend the therapist's expertise, there still exists a view in some clinical quarters that any technology serves to subvert the clinical relationship.

***Unrealistic expectations***. First-time SG users often experience unrealistic expectations, mostly based on overhyped media representations. This can lead to some disappointment when starting to use SG.

***Poor academic and professional acceptance***. SG are viewed by many researchers and clinicians as expensive toys gaining no scientific and clinical credibility. The perception of SG in therapy and rehabilitation contexts is slowly starting to change, and SG are gradually being accepted as tools providing new treatment options. This perception change is partly due to the increased number of articles published in mainstream journals, along with conferences dedicated to this topic. However, there is still a consistent part of the scientific community that derides the idea of using SG for scientific and clinical purposes. Controlled clinical trials will be necessary to evaluate the effectiveness of SG targeting dementia-related disorders, and to increase the academic and professional acceptance.

***Technophobia***. The fear or dislike of ICT is still common in many elderly people, as well as in some of their family caregivers and healthcare professionals. This can make it difficult to employ SG as assessment and rehabilitation tools for some of the present generation of elderly people.

***Serious Game design***. Participants working on the SG design session of the first round were asked to provide a definition of SG, and proposed the following: “A SG is a solution that combines entertainment and motivation to facilitate learning and social bonding in the context of an activity.” Furthermore, they discussed about two SG currently under development at the CoBtek research unit: “Panic at the Nursing Home” devoted to the training of nursing home professional staff, and “battle ship” (http://www.azagame.fr/) devoted to the physical and cognitive training of patients with ADRD. Finally, participants were asked to generate ideas for a SG adapted to people with ADRD. They proposed the following games: (1) a cultural game taking place in a museum, where players can navigate different collections of artifacts, and in each location they are asked questions and play games to train their memory and executive functions; (2) a social quiz game (similar to the board game “Trivial Pursuit”) to be played by people with ADRD together with young people, with the aim of training memory, and promote inter-generational bonding.

***Recommendations***. Participants in the recommendation session of round 1 were asked to:

◦ Respond to 6 questions (see Table 1); after a brainstorming with a free response format, participants were presented with a list of items for each question, and asked to rate the importance of each item on a 0–3 scale (see Methods).
◦ Comment and discuss practical recommendations for the development and use of SG in people with ADRD.

### Questions

**Figure 1:**
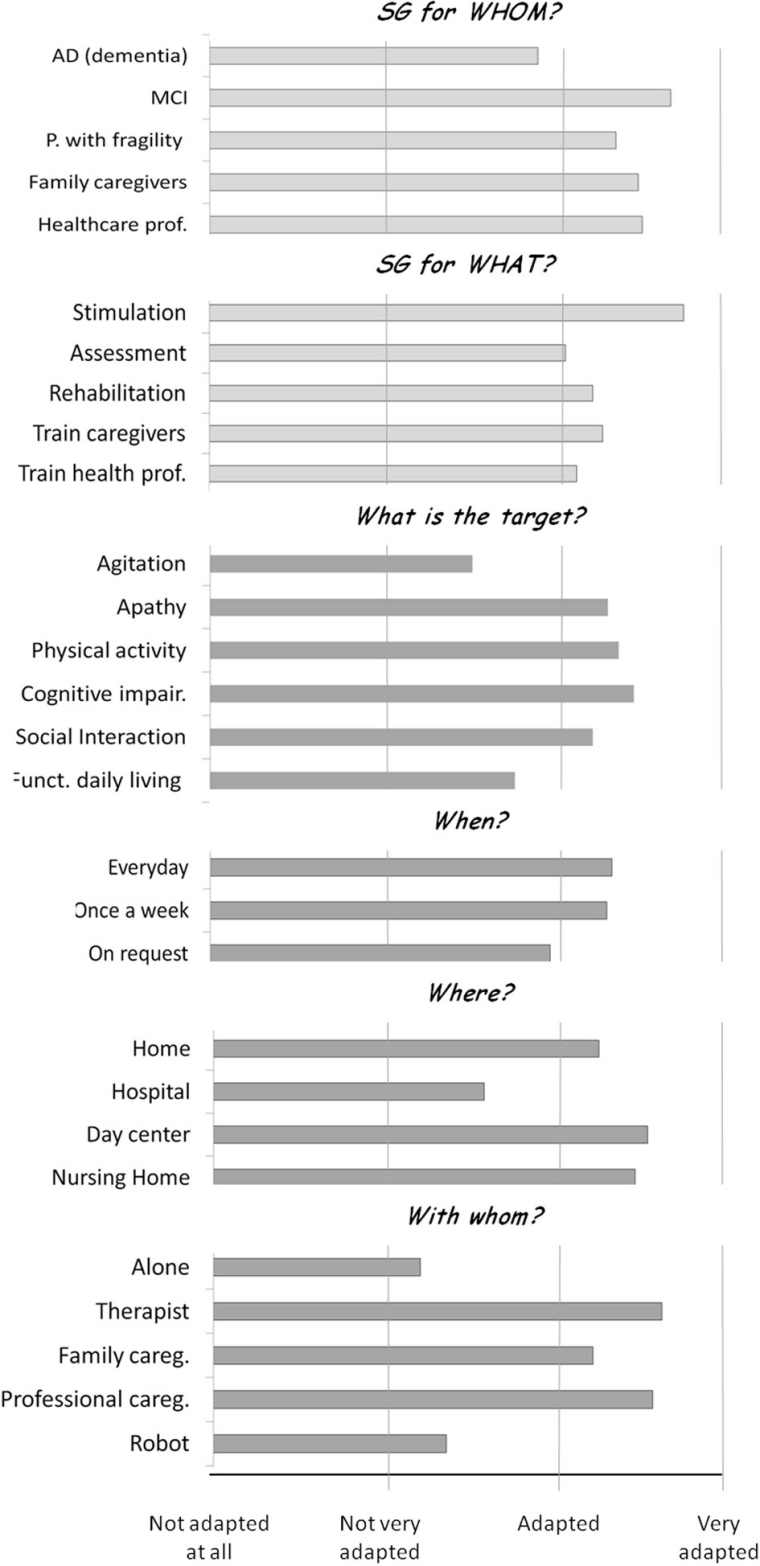
**Results of the recommendation questions**. Mean ratings provided by participants of round 1 of the recommendation session and participants of the online survey to the two general questions (light gray) and the four questions focused on ADRD (dark gray). Rating scale: 0–3 scale (0, not adapted at all; 1, not very adapted; 2, adapted; 3, very adapted).

The results of the questions (mean scores of the ratings assigned to each item, collapsed across participants from round 1 and from the online survey) are reported in Figure 1. The additional elements emerged during the brainstorming of round 1, and the additional comments provided by participants of round 2 are reported below.

#### Generals questions

- SG for whom? i.e., what should be the target population for SG?

Based on a literature review (e.g., McCallum and Boletsis, 2013), and on the authors' clinical expertise, participants were asked to rate if SG are adapted to the following categories of target people: AD (dementia stage), MCI, people with frailty, family caregivers and health professionals.

The results of the ratings showed that SG are considered between “adapted” and “very adapted” to: people with MCI, people with frailty syndrome, and family and professional caregivers. The highest score was assigned to the use of SG for people with MCI. SG were rated between “not very adapted” and “adapted” to people with AD and other dementia-related disorders. The experts in round 2 agreed that most of the SG currently available on the market are not very adapted to AD and dementia-related disorders, but suggested that designing SG for these target populations, though challenging, should be considered a priority. Furthermore, they highlighted that professional caregivers should be sensitized to the opportunity to use SG for dementia-related disorders. SG may also be employed as an engagement tool to train caregivers and provide them with more “active” dementia-related information, as opposed to the traditional standard training methods. The additional categories of target populations proposed during the brainstorming of round 1 included: all people; people with handicap, people suffering with phobias, autism, eating disorders, sleep disorders, depression, apathy, behavioral disturbances, chronic illnesses, people with reduced autonomy, people alone, and isolated, and old/new friends of people with impairments.

- SG for what? i.e., Why is it interesting to use the SG?

Participants were asked to rate if SG are adapted for the following purposes: to stimulate, assess and rehabilitate the patients, and to train caregivers and healthcare professionals. The results of the ratings showed that SG are considered between “adapted” and “very adapted” for patient stimulation, assessment, and rehabilitation and for the training of caregivers and healthcare professionals. The highest score was assigned to the use of SG for patients' stimulation. The additional categories proposed during the brainstorming of round 1 included: for everything, to have fun, to share and communicate, to help to address fears and phobias, to train in using new technologies, to help to accomplish everyday tasks, to educate, to improve the sense of self-efficacy, to work, for prevention purposes, and to reduce the cost of healthcare for the society, social security, and associations.

#### Questions focused on AD, related disorders, and frailty

- Why should SG be employed with patients with ADRD? What is the clinical target?

Participants were asked to rate whether SG are important to target AD patients: agitation, apathy, physical activity, cognitive impairment, impaired/reduced social interaction, and reduced functionality in activities of daily living. These categories were selected as they represent some of the most common symptoms of dementia, occurring in up to 90% of people with ADRD (Cerejeira et al., 2012). As the terms “agitation,” “apathy,” and “functionality in activities of daily living” are terms that can be employed with different meanings in different clinical populations, we will briefly clarify their meaning in the context of ADRD.

Agitation is a neuropsychiatric symptom defined as “inappropriate verbal, vocal, or motor activity that is not judged by an outside observer to result directly from the needs or confusion of the agitated individual” (Cohen-Mansfield et al., 2010). Confusion, discomfort, and unmet needs may underscore agitation, but the outward behavioral expression is usually the result of a need not being addressed (Cohen-Mansfield, 1999) as well as an attempt to overcome passivity (Yu et al., 2006). Those unmeet needs, often present in AD patients, particularly institutionalized, are addressed quite successfully by non-pharmacological interventions such as music therapy (Gerdner, 2000; Remington, 2000) and physical activity (Alessi et al., 1999; Buettner and Fitzsimmons, 2002), and thus agitation should be susceptible to improvement through the use of SG.

Apathy is one of the most common neuropsychiatric symptoms of AD, occurring in almost 65% of dementia patients (Ferri et al., 2005; König et al., 2014) being associated with a higher degree of global functional impairment (Doron et al., 2013) and therefore loss of autonomy in activities of daily living (Boyle et al., 2003; Scarpini et al., 2003; Lechowski et al., 2009). Marin (1991) defined it as a lack of motivation, interest, emotion, or feeling. Recently, Robert et al. (2009) proposed a consensus definition in terms of a set of diagnostic criteria for apathy in Alzheimer's disease (AD). According to those criteria, an apathy diagnosis should meet the following requirements: first, the core feature of apathy, diminished motivation, must be present for at least 4 weeks; second two of the three dimensions of apathy (reduced goal-directed behavior, goal-directed cognitive activity, and emotions) must be present; third there should be identifiable functional impairments attributable to apathy. Most recent models consider apathy particularly in terms of an “absence of responsiveness to stimuli as demonstrated by lack of self-initiated action” (Andersen et al., 2004; Levy, 2012). Due to their playful nature, SG may be particularly adapted to target apathy deficits.

Finally, impairment in functionality in activities of daily living represents a major diagnostic requirement for AD (American Psychiatric Association, 1994). Several studies have investigated the link between cognitive functioning and daily functioning, both necessary to live autonomously. Cognitive domains such as memory, executive functioning, visuospatial functions, and object perception have all been found to correlate with activities of daily living impairment (Perry and Hodges, 2000; Glosser et al., 2002; Plehn et al., 2004; Jefferson et al., 2006; Tomaszewski Farias et al., 2009). Those activities can be divided into basic activities of daily living such as self-maintenance skills, and Instrumental activities of daily living (IADL) such as preparing a meal and handling finances (Lawton and Brody, 1969). Complex IADL activities are sensitive to cognitive decline in early stages of AD, whereas basic activities remains preserved until later and more severe stages of the disease (Stern et al., 1990; Barberger-Gateau et al., 2000; Wilms et al., 2000; Peres et al., 2006). SG could be employed to train those activities, in order to promote a longer independent life at home.

The results of the ratings showed that SG are considered between “important” and “very important” to target apathy, motor activity, cognitive impairments, and diminished social interaction, with the highest score assigned to cognitive impairment. SG were rated between “not very important” and “important” to target agitation and reduced functionality in activities of daily living. The experts during round 2 suggested that SG could be further employed to target agitation. As seen above, very often agitation in people with dementia-related disorders is caused by the desire to overcome passitivity (Yu et al., 2006). SG may represent an interesting and fun activity that could be possibly linked to an intervention: for instance, showing old pictures, or listening to well-known songs may have a calming effect. The experts also suggested that SG may be employed in order to improve patients' functionality in activities of daily living, especially for patients with MCI and patients in the AD pre-clinical stages. Importantly, SG should target depression and anxiety in AD and dementia-related disorders. Finally, the experts advised that SG should start to target a single ability/problem (e.g., the fear of falling down) and then extend progressively to other abilities (e.g., memory). The additional categories proposed during the brainstorming of round 1 includes: to create an intergenerational link, to improve mood, to combine different targets (e.g., physical + cognitive activity), to teach new abilities, to give personalized information on therapeutic goals, to help the patient to orient (knowing the time and day, etc.), to improve sleep and eating disorders, to promote cultural aspects.

- When (how frequently) should SG be used in patients with ADRD?

Participants were asked to rate if SG are adapted to be used everyday, once a week, and on request. The results of the ratings showed that SG are considered between “adapted” and “very adapted” to be used every day, once a week, and on request, with the highest score assigned to the use of SG every day. The additional categories proposed during the brainstorming of round 1 included: frequently and regularly.

- Where should SG be used for patients with ADRD

Participants were asked to rate if SG are adapted to be used at home, at the hospital, in the day centers and at the nursing homes. The results of the ratings showed that SG are considered between “adapted” and “very adapted” to be used at home, in the nursing homes, and in the day centers, with the highest score assigned to the use of SG in day centers. SG were rated between “not very adapted” and “adapted” to be used at the hospital. An additional category proposed during the brainstorming of round 1 was: in the waiting rooms

- Whom should patients with ADRD play SG with?

Participants were asked to rate whether SG are adapted to be used by the patient alone, and by the patient together with a therapist, a family caregiver, a professional caregiver, and a robot. The results of the ratings showed that SG are considered between “adapted” and “very adapted” to be used with a therapist, a professional caregiver and a family caregiver, with the highest score assigned to the use of SG with the therapist. SG were rated between “not very adapted” and “adapted” to be used alone and with a robot. The experts in round 2 added that SG could be played by the patient alone in the preclinical stages and the early clinical stages, to decrease the time, burden and effort the caregiver dedicates to the patient. The additional categories proposed during the brainstorming of round 1 included: in a group, with nurses.

### Practical recommendations for the development of SG targeting ADRD

Participants of the recommendation session of the first round were also presented with a list of recommendations for the development of SG targeting ADRD gathered from a literature review of the field (e.g., Bouchard et al., 2012; Fua et al., 2013). These recommendations are reported below, together with the comments and integrations provided by the participants of the recommendation session (first round), and the additional comments provided during the second round.

Based on literature review, in order to be used for assessment and rehabilitation purposes of dementia—related disorders, SG should:

1. Keep track of the patient's cognitive abilities. The game should produce an in-game estimation of the patient's cognitive abilities. This would allow the clinician, caregiver and patient to assess the impact of the game on cognitive performance, and could be used to adapt the level of game difficulty to the actual performance achieved.
2. Determine an appropriate number of steps for the challenge. Each challenge should be completed in a correct number of steps. A high enough number of steps would correctly train the cognitive abilities of the patients. However, too many steps could overload them and lower the benefits of the game.
3. Keep the player in his “flow zone.” Flow represents the feeling of complete and energized focus in an activity, with a high level of enjoyment and fulfillment. Maintaining flow will make the game more enjoyable, improving the learning experience.
4. Promote naturalistic interactions. Employing naturalistic interactions should allow a significant reduction of the learning time, and thus optimize the effects of the game experience.
5. Use user-friendly interface for home-based exercises. Design choices should be made in a way to facilitate home-based rehabilitation training.
6. Take advantage of the multimodal aspect. It is important to train sensory and motor modalities at the same time. When possible, multi-sensory interactions should be introduced both as input and output.
7. Take into account the impairments of the users. It is crucial to consider that ageing and dementia have important effects on the sensory, motor, and cognitive system. Patients with dementia can suffer from visual and hearing problems, and have troubles with many aspects of memory (e.g., working memory, semantic memory, episodic memory, prospective memory). Furthermore, they can show a decline in the processing speed, have impairments in some executive functions (such as reasoning) and visuo-spatial abilities, and show some motor impairments. SG should take these impairments into account, for instance, keep the visual scenes as simple as possible, provide instructions, and cues repeatedly and at the right time (possibly using different modalities, e.g., auditory and visual cues), minimize actions requiring a fast response and requiring complicated movements, and so on (see Fua et al., 2013 for a review).
  The participants of the first round of the IA workshop confirmed that all the recommendations reported above are relevant for the application of SG to people with ADRD. In addition, the following recommendation emerged:
8. Take into account the social and cultural background of the user. To promote ecological interactions, it is necessary to situate the game in the correct physical place (e.g., a city well known to the user) and cultural environment.

## Discussion and future research directions

The recommendations and analyses reported in the present paper have been carried out by and for healthcare professionals, patient, and family associations' representatives, engineers, and companies involved in the development of SG. Even if the overestimated expectations, frequently presented by the media, are far from being achieved, our SWOT analysis showed that SG can be considered as useful tools for professionals involved in the care of patients suffering from ADRD. However, more interdisciplinary work should be done in order to create SG specifically targeting these populations. The employment of SG in the domain of rehabilitation of ADRD is still in an early phase of development, characterized by some early encouraging research results. The many listed strengths provide a justification for continuing in this direction. Weaknesses do exist (in particular limitations concerning the difficulty that patients experience while using SG interfaces), as well as some threats, but none are terminal, especially when addressed in collaboration with the different partners involved in the development of SG for rehabilitation. In order to acquire more academic and professional credibility and acceptance, the field of SG would need to invest more in research. Specifically, researchers need to start collecting incremental data over numerous small-scale studies to test and evolve usability, usefulness, and access of SG targeting people with dementia-related disorders. At the same time, ethical, professional, and cost/benefit issues need to be considered and addressed.

The field will need to face a number of challenges, which will benefit from the multidisciplinary collaboration between engineers, researchers, clinicians, healthcare professionals, patients, and family caregivers. For instance, it would be important for the companies and manufactures involved in the design and commercialization of SG to work closely not only with people in the ICT domain, but also with people coming from the health domain and with researchers. National and international agencies (such as the CNR in France—Centre national de reference en Santé) aiming to facilitate the communication and exchange between scientific community and private companies, may play a major role in driving this interdisciplinary exchange.

The recommendations gathered during the IA workshop addressed several abilities and deficits that SG may target in people with ADRD, as well as different ways to employ SG based on the patients' impairments, and may be used as guidelines to design SG specifically adapted to people suffering from these disorders. A limitation of the present work is that the participants of the IA workshop were asked to provide recommendations concerning the development and use of SG for people with frailty and ADRD, but we did not specify at which stage of the disease progression. As neuro-degenerative diseases are characterized by different stages of impairment, future investigations should better clarify which recommendations are adapted to the different disease stages.

### Conflict of interest statement

Philippe H. Robert received research grants from: EEC, ANR, CoVEA, Eisai, and Lundbeck. He also received honorarium from Lundbeck, Lilly, Novartis, Jansen, Roche, Ipsen. François Bremond has declared associations with the following companies: Genious, Interactive4D, SolarGames. Stéphane Nave is full time employee at Hoffmann La Roche, Basel. Jean M. Orgogozo is a consultant for Novartis-Bases and is a corporate partner of Pharnext Biotech, Paris. The other authors declare that the research was conducted in the absence of any commercial or financial relationships that could be construed as a potential conflict of interest.
